# Ultrastructure of plastids serves as reliable abiotic and biotic stress marker

**DOI:** 10.1371/journal.pone.0214811

**Published:** 2019-04-04

**Authors:** Bernd Zechmann

**Affiliations:** Center for Microscopy and Imaging, Baylor University, Waco, Texas, United States of America; Chinese University of Hong Kong, HONG KONG

## Abstract

Plastids perform many essential functions in plant metabolism including photosynthesis, synthesis of metabolites, and stress signaling. The most prominent type in green leaves is the chloroplast which contains thylakoids, plastoglobules, and starch. As these structures are closely linked to the metabolism of chloroplasts, changes during plant growth and development and during environmental stress situations are likely to occur. The aim of this study was to characterize changes in size and ultrastructure of chloroplast on cross-sections of leaves during high light stress, Botrytis infection, and dark induced senescence by quantitative transmission electron microscopy (TEM).The size of chloroplasts on cross sections of leaves decreased significantly when plants were subject to high light (49%), Botrytis infection (58%), and senescence (71%). The number of chloroplasts on cross sections of the palisade cell layer and spongy parenchyma, respectively, decreased significantly in plants exposed to high light conditions (48% and 29%), infected with Botrytis (48% and 46%), and during senescence (78% and 80%). Thylakoids on cross-sections of chloroplasts decreased significantly in plants exposed to high light (22%), inoculated with *Botrytis cinerea* (36%), and senescence (51%). This correlated with a massive increase in plastoglobules on cross-sections of chloroplasts of 88%, 2,306% and 19,617%, respectively. Starch contents on cross sections of chloroplasts were completely diminished in all three stress scenarios. These results demonstrate that the decrease in the number and size of chloroplasts is a reliable stress marker in plants during abiotic and biotic stress situations which can be easily detected with a light microscope. Further, lack of starch, the occurrence of large plastoglobules and decrease in thylakoids can also be regarded as reliable stress marker in plants which can be detected by TEM.

## Introduction

The plastid is an organelle of great significance for plants. It performs photosynthesis by utilizing carbon dioxide and water to synthesize different chemical components that are converted by the plant into sugars and other biomolecules [1,2]. It acts as storage compartments for glucose in the form of starch [3] and other biomolecules such as lipids, amino and nucleic acids [2]. It is involved in plant metabolism by synthesizing phytohormones and other secondary metabolites [2,4]. It is also involved in sensing and signaling stress to other cell compartments which can then lead to adaptations of growth and development of the plant [4,5,6,7].

**Fig 1:**
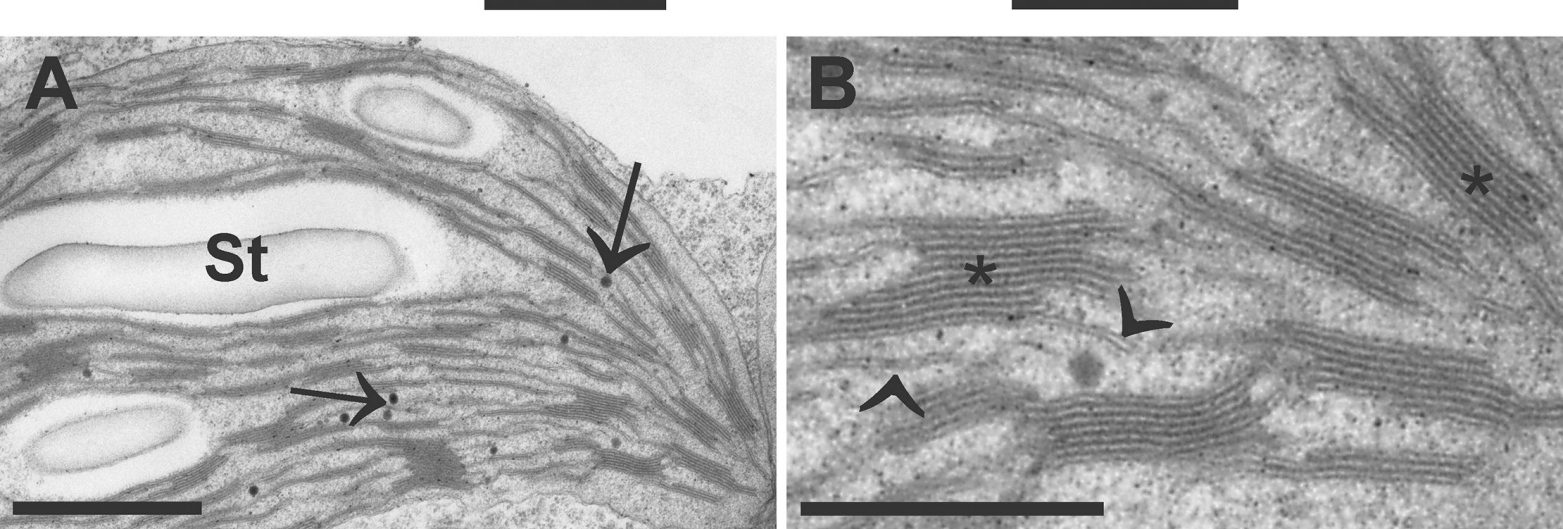
Ultrastructure of a chloroplast imaged by transmission electron microscopy. Chloroplast contains large starch grains (St), thlylakoids in the form of single membranes (arrowheads) or grana stacks (asterisks), and plastoglobules (arrows) in the dense stroma. Bars = 1 μm in A and 0.5 μm in B.

Form, size, and ultrastructure of plastids vary greatly between different developmental and physiological states of the plant, the function of the organ, and the tissue [8]. In green leaves for example the most prominent form of the plastid is the chloroplast which contains fine structures such as thylakoids, starch, stroma, and plastoglobules (Fig 1). The stroma of the chloroplast is separated from the cytosol by a double membrane and contains DNA, RNA, and ribosomes. Within the stroma lay thylakoids, which are membranes that enclose the intrathylakoidal space and either appear as single stacks or grana stacks [9,10,11]. They contain pigments, enzymes, and other biomolecules involved in the light reaction during photosynthesis. Depending on the amount of glucose produced by the chloroplast and needed inside the cell, chloroplasts may or may not contain starch grains [3]. They appear as electron translucent round or ellipsoid grains inside the chloroplasts and are not surrounded by a membrane. They usually shrink during chemical fixation and are therefore surrounded by an electron translucent area that appears brighter than the starch grain itself (Fig 1). Plastoglobules are electron opaque round objects inside the plastids and are either connected to the outer membrane of thylakoids or occur separated from thylakoids inside the stroma [12, 13]. They consist of lipids that are similar to those found in thylakoids, they contain enzymes that synthesize lipids in thylakoids, and they contain metabolites which are involved in plastid development. They are involved in metabolite synthesis, repair, and disposal which are essential during plastid development, aging and adaptation to stress [12, 13].

In this study ultrastructural changes of chloroplasts were compared between plants exposed to abiotic stress (high light stress), biotic stress (*Botrytis cinerea* infection), and plants that went through dark-induced senescence. The exposure of plants to high light leads to the closure of stomata which decreases CO_2_ levels inside the leaf and subsequently leads to disturbances of photosynthesis and to oxidative stress in illuminated chloroplasts [14, 15, 16, 17]. An accumulation of hydrogen peroxide (H_2_O_2_) in mitochondria and chloroplasts has been observed in plants infected with the fungal pathogen *Botrytis cinerea* [18]. The oxidative burst leads to the development of necrosis and disintegration of the ultrastructure of infected tissue which restricts the spread of the diseases [18, 19, 20]. Dark induced senescence is characterized by loss of proteins, degeneration of chlorophyll and nucleic acid which goes hand in hand with a controlled breakdown of cell and organelle structures [21, 22]. Considering the above described metabolic changes it is not surprising that the ultrastructure of chloroplasts goes through dramatic changes during abiotic and biotic stress. Such changes include changes in the number and size of plastoglobules during abiotic stress such as drought [23,24,25], excess light conditions [26,27,28], and biotic stress such as infection of plants with pathogens [29, 30, 31, 32, 33]. An increase in the number of plastoglobules has been observed during senescence [21,22,34,35]. A decrease in the size of starch grains in chloroplasts was observed during drought [23,24] whereas an increase and or decrease was found during biotic stress such as infection of plants with viral pathogens [36]. As the above described studies have been performed with different plants species under different environmental conditions with different outcomes it is difficult to precisely evaluate how abiotic and biotic stress conditions affect chloroplast ultrastructure and if such changes can serve as reliable marker for stress.

Thus, the aim of this study was to investigate and quantify changes in the ultrastructure of chloroplasts on cross-sections of *Arabidopsis thaliana* leaves during senescence, abiotic and biotic stress in order to get a deeper insight into how metabolic alterations observed during these studies affect the ultrastructure of chloroplasts. The study was also aimed to dissect which ultrastructural feature in chloroplast would be affected most by the different stress situations and would therefore be best suited as stress marker.

## Materials and methods

### Plant material

After stratification for 4 d at 4°C seeds of *Arabidopsis thaliana* [L.] Heynh. Ecotype Columbia (Col-0) were grown in a growth camber with 8:16 h, light:dark period at 22:18°C. The relative humidity was 60% and the plants were kept at 100% relative soil water content. Control plants were kept at a light intensity between 120 and 140 μmol m^-2^ s^-1^ (lower and upper leaves, respectively). High light stress was induced by treating 6-week-old plants grown in soil with 1,500 μmol m^-2^ s^-1^ of light (Plug and Grow, 6400 K, white/blue spectrum; Agriculture Trading AG, Walenstadt, Switzerland) for 2 weeks. Dark induced senescence in 6-week-old plants grown in soil was induced by covering fully developed leaves from the 3^rd^ rosette with aluminum foil for 10 days. Control plants were kept at a light intensity between 120 and 140 μmol m^-2^ s^-1^. For Botrytis inoculation leaves of the 3^rd^ rosette of 8 week-old plants were cut off and placed into petri dishes containing filter paper wetted with sterile water. A 50 ml drop of a *Botrytis cinerea* (strain: Bo510) spore suspension was placed onto the abaxial side of each leaf (approximately 1x10^5^ spores ml^-1^) for 96 h. Spores were preincubated in 3 g L^-1^ Gamborg’s B5 medium (in 10 mM KH2PO4 buffer, pH 7) supplemented with 25 mM glucose for 2 h to stimulate germination. Control leaves were treated likewise but without spores in the medium. Petri dishes were sealed and stored at light conditions described above.

### Electron microscopy

For transmission electron microscopy small pieces of leaves (about 1mm^2^) were cut out in 2.5% glutardialdehyde in 0.06M Sørensen phosphate buffer at pH 7.2 on a modeling wax plate. Leaves were then transferred into glass vials and fixed for 90 min in the above-mentioned solution. For ultrastructural analysis samples were then rinsed in 0.06 M Sørensen phosphate buffer (4 times for 15 min each) and post-fixed in 1% osmium tetroxide in 0.06 M Sørensen phosphate buffer for 90 min at RT. The samples were then dehydrated in a graded series of increasing concentrations of acetone (50%, 70%, 90%, and 100%). Pure acetone was then exchanged for propylene oxide and the specimens were gradually infiltrated with increasing concentrations of Agar 100 epoxy resin (30%, 60%, and 100%) mixed with propylene oxide for a minimum of 3 h per step. Samples were finally embedded in pure, fresh Agar 100 epoxy resin (Agar Scientific Ltd, Stansted, UK) and polymerized at 60°C for 48 h. Ultrathin sections (80 nm) were cut with a Reichert Ultracut S ultramicrotome (Leica Microsystems, Vienna, Austria), post-stained with lead citrate (1% dissolved in 0.6 M NaOH) and uranyl-acetate (2% dissolved in aqua bidest) for 15 min. Sections were then observed in a Philips CM10 TEM.

### Determination of chloroplast number and their fine structures

Changes in the number of chloroplasts and their ultrastructure were evaluated according to Zechmann et al. [30] by investigating four different leaf samples from treated and control plants. An Olympus AX70 light microscope (Olympus, Life and Material Science Europa GmbH, Hamburg, Germany) with a 40x objective lens (n.a. 1.35) was used to determine the number of sectioned chloroplasts in the palisade cell layer and the spongy parenchyma by counting the chloroplasts per cell on 4 semithin cross-sections (3 μm) for each replicate sample. A minimum of 100 cells per leaf type were examined to calculate the number of sectioned chloroplasts in the cells. Ultrathin sections were investigated with the TEM to determine changes in the ultrastructure of the chloroplasts including the thylakoid-system, starch grains, and plastoglobules. The areas (μm^2^) of these structures were then measured as digital images using the program Optimas 6.5.1 (BioScan Corp.) by manually tracing the perimeter of thylakoids, plastoglobules, starch grains, and the perimeter of the whole chloroplast. The areas of stroma were determined by subtracting the areas of thylakoids, starch, and plastoglobules from the total area of the chloroplasts. Chloroplast fine structures (stroma, thylakoids, starch, and plastoglobules) were then converted into relative areas (% of total chloroplast area) to allow comparisons between different treatments. A minimum of 20 sectioned chloroplasts from at least 10 different cells from four different samples per treatment were examined. The obtained data were statistically evaluated with SPSS Statistics (IBM Corp. New York, USA) by applying the Mann–Whitney U test.

## Results

**Fig 2:**
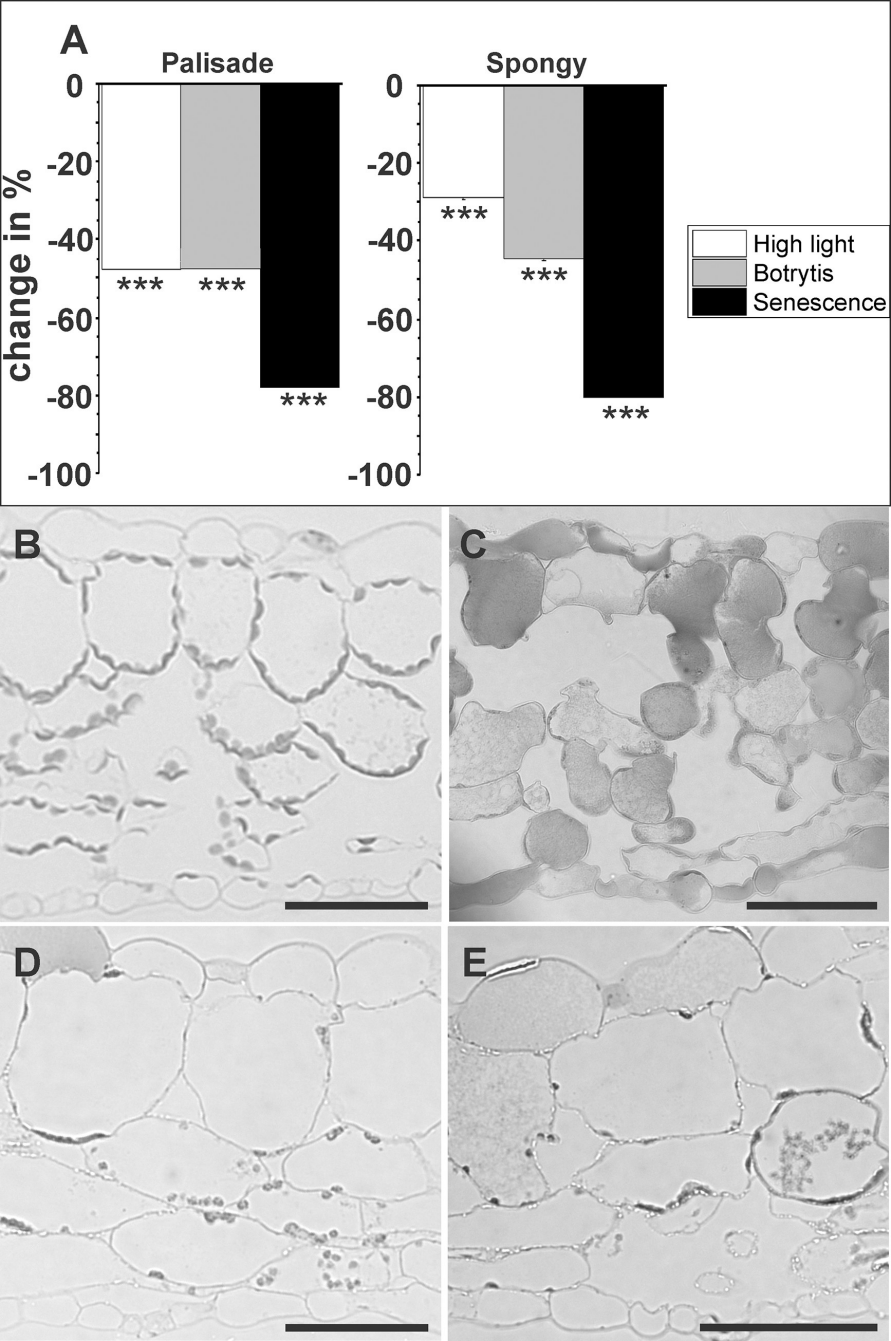
Changes (%) in the number of plastids in the palisade cell layer and the spongy parenchyma induced by high light, Botrytis infection, and senescence. Data in graphs (A) are means with standard errors determined by image analysis on semi-thin cross sections of leaves according to Table 1. Plastids per sectioned cell (n>100 per treatment) were counted with a light microscope in four replicate semi-thin sections of control plants (B), plants grown under high light conditions of 1,500 μmol m^-2^ s^-1^ (C), inoculated with Botrytis (D), and after dark induced senescence (E). Significant differences were calculated using the Mann- Whitney U test; *** indicate a significance at a level of confidence of p<0.001. Bars = 100 μm.

The number of chloroplasts on cross-sections of the palisade cell layer decreased significantly in plants exposed to high light conditions and infected with Botrytis (both 48%). Seventy eight % less chloroplasts were detected on cross sections of leaves exposed to dark induced senescence. A similar situation was found in the spongy parenchyma where the number of chloroplasts decreased significantly on cross-sections of leaves exposed to high light conditions (29%), infected with Botrytis (46%), and during senescence (80%) (Fig 2, Table 1).

**Table 1:** Number (#), absolute area (in μm^2^), and relative area (in %) of chloroplasts and fine structures on cross sections of control plants and plants grown under high light conditions, inoculated with Botrytis, and during senescence.

	Control	High Light	Control	Botrytis	Control	Senescence
**# Chloroplasts** **Palisade (n = 100 each)**	8.7 ± 0.4	4.5 ± 0.4***	17.3 ± 0.4	9.1 ± 0.4***	12.9 ± 0.2	2.8 ± 0.2***
**# Chloroplasts Spongy (n = 100 each)**	6.2 ± 0.3	4.4 ± 0.3***	7.9 ± 0.3	4.3 ± 0.3***	9.4 ± 0.2	1.9 ± 0.2***
**Chloroplast size in** μ**m**^**2**^ **(n = 30 each)**	9.4 ± 1.0	4.8 ± 0.5**	7.3 ± 0.7	3.1 ± 0.3***	10.4 ± 0.5	3.0 ± 0.2***
**% Stroma**	65.8 ± 2.6	82.7 ± 2.6**	62.0 ± 0.4	69.0 ± 1.3^ns^	55.4 ± 1.7	57.2 ± 1.7^ns^
**% Thylakoids**	20.7 ± 1.1	16.2 ± 1.1**	24.5 ± 0.8	15.6 ± 0.7***	27.2 ± 1.0	13.2 ± 1.0***
**% Starch**	12.9 ± 2.7	0.0***	12.9 ± 1.6	0.0***	17.2 ± 1.6	0.0***
**% Plastoglobules**	0.6 ± 0.0	1.1 ± 0.0***	0.6 ± 0.0	15.4 ± 0.1***	0.2 ± 0.0	29.6 ± 0.1***

**Fig 3:**
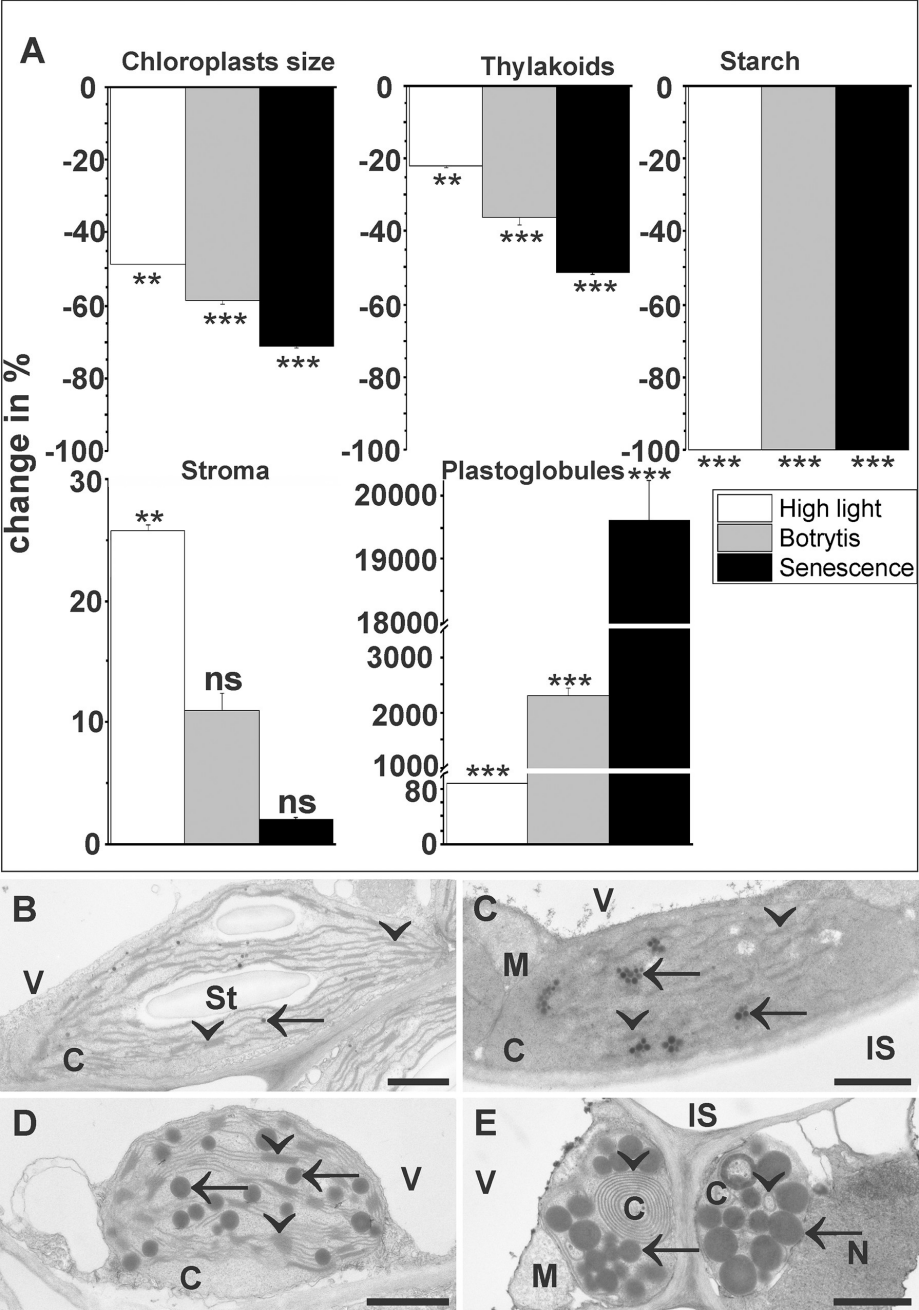
Changes (%) of the ultrastructure of plastids induced by high light, Botrytis infection, and senescence. Data in graphs (A) are means with standard errors determined by image analysis on ultra-thin cross sections of leaves according to Table 1. Changes of the area of plastid fine structures were determined by TEM and image analysis of ultra-thin sections of control plants (B), plants grown under high light conditions of 1,500 μmol m^-2^ s^-1^ (C), inoculated with Botrytis (D), and after dark induced senescence (E). Significant differences were calculated using the Mann- Whitney U test; ** and *** indicate a significance at a level of confidence of p<0.01 and p<0.001. C = chloroplasts, IS = intercellular space, M = mitochondria, N = nucleus, St = starch, V = vacuoles. Arrows = plastoglobules, Arrowheads = thylakoids. Bars = 1 μm.

Data are means with standard errors and represent the number (#) of chloroplasts in palisade and spongy parenchyma on semi-thin sections of leaves, the area of chloroplasts in μm^2^, and the relative areas in percent of stroma, thylakoids, starch, and plastoglobules on ultra-thin sections of cells. These data have been used to calculate the changes displayed in Figs 2 and 3. The number of chloroplasts was determined on at least 100 sectioned cells for each treatment by light microscopy while the size and ultrastructure of chloroplasts was determined on at least 30 sectioned chloroplasts for each treatment by TEM. Significant differences between stressed plants and the according controls were calculated using the Mann- Whitney U test

** and *** indicate a significance at a level of confidence of p<0.01 and p<0.001; ns = not significant different.

The ultrastructure of chloroplasts showed significant changes after the exposure to abiotic and biotic stress. The size of chloroplasts on ultra-thin cross-section decreased significantly when plants were subject to high light (49%), Botrytis infection (58%), and senescence (71%). Thylakoid areas decreased significantly on cross-sections of chloroplasts exposed to high light (22%), inoculated with *Botrytis cinerea* (36%), and during dark-induced senescence (51%). A 100% decrease of starch was observed during all stress scenarios (Fig 3). The area of stroma significantly increased in cross-sections of chloroplasts of plants exposed to high light conditions (26%) and remained statistically unchanged in plants during Botrytis infection and dark induced senescence (Fig 3). Plastoglobules showed a strong increase of 88% on cross-sections of chloroplasts from plants exposed to high light, 2,306% in plants inoculated with *Botrytis cinerea*, and 19,617% in plants during dark-induced senescence (Fig 3, Table 1).

## Discussion

High light stress, Botrytis infection, and senescence had similar effects on the ultrastructure of chloroplasts. In all three cases the number and size of chloroplasts, areas of thylakoids, and starch significantly decreased on cross-sections of leaves. Similar effects have also been observed in other plants under similar circumstances [37, 38, 39]. During *Botrytis cinerea* infection changes in size and ultrastructure of chloroplasts can be attributed to the accumulation of reactive oxyigen species (ROS), especially hydrogen peroxide, in this organelle [18,19] which activates apoptotic like programmed cell death (PCD) [20,40]. These events are especially pronounced at the final phases of Botrytis infection when the fungus switches from the biotrophic to necrotrophic life style and the plant reacts with cell death to block fungal growth [19,20,40]. Similar effects on chloroplast size, number, and ultrastructure were also found in cross-sections of leaves during dark-induced senescence, which is a highly regulated process mediated by a genetic program which remobilizes nutrients from the aging organs to support growth and development of younger ones or to store remobilized nutrients in seeds or perennial tissue [12,41]. The accumulation of ROS, especially hydrogen peroxide, in chloroplasts seems to be the main trigger for PCD during dark induced senescence which leads to chloroplast degradation, the reduction of photosynthetic efficiency, and a decrease of chlorophyll [42,43]. During Botrytis infection and senescence chloroplasts are considered to be the primary source and target of PCD as they host genes involved in PCD (e.g. accelerated death 2), can release cytochrome f into the cytosol which triggers PCD, and produce ROS which destroys membranes and proteins and induce hypersensitive response [40]. Thus, it is not surprising that thylakoids were strongly reduced in cross-sections of chloroplasts exposed to Botrytis infection and during senescence in this study. ROS damage thylakoids through a process called lipid peroxidation which compromises membrane leakage and integrity [44,45]. Additionally, the accumulation of ROS in chloroplasts during Botrytis infection and senescence can activate gene expression leading to PCD [46,47].

The exposure of plants to high light led to similar changes in chloroplast size, number, and ultrastructure on cross-sections of leaves as observed during Botrytis infection and senescence. High light stress leads to photodamage in chloroplast through the accumulation of ROS which oxidize membranes and inhibit repair of photodamaged photosystem II [45,47,48]. Thus, a decrease in chloroplast size and changes in the ultrastructure such as degeneration of thylakoids seem to be a logical consequence. The degradation of thylakoids, observed in this study, correlated with an increase in the size of plastoglobules on cross-sections of chloroplasts, which is not surprising as they are physically connected to the outer membrane of thylakoids and appear to participate in the formation and degradation of thylakoids during plant growth and development, stress, and senescence [12,13]. The breakdown of chlorophyll and subsequently thylakoids is a commonly observed reaction of plants to high light stress [49], *Botrytis cinerea* infection [39], and during senescence [37,38]. Under these conditions, components of thylakoids such as carotenoids, fatty acids, and prenyl quinones are deposited into plastoglobules [50,51]. Additionally, it has been proposed that catabolic products released from thylakoids during senescence such as pheophytin pheophorbide hydrolase, triacylglycerol, fatty acid phytylester, get deposited into plastoglobules [12,13]. Thus, the massive increase in the size of plastoglobules in cross-sections of chloroplasts in this study can be attributed to the deposition of thylakoid membrane components which were degraded during the exposure of plants to high light, Botrytis infection, and senescence.

It is also important to mention that starch contents in cross-sections of chloroplasts were strongly decreased in all three investigated stress scenarios. During high light conditions plants immediately react with the remobilization of starch which increases soluble sugars levels. This reaction is aimed to provide energy and carbon to the plant as high light conditions lead to photoinhibition and decreased rates of carbon fixation [52,53,54]. During senescence plants react with the hydrolysis of starch and accumulation of soluble sugars [43]. Since leaf senescence can be stimulated in the presence of glucose [55,56] and mutants with impaired sugar sensing capabilities show delayed senescence [57] it seems that the accumulation of soluble sugars is an important factor for the induction of senescence. Thus, it seems that the decrease of starch contents in cross-sections of chloroplasts in all three investigated scenarios is caused by reduction of starch to soluble sugars and by the degeneration of thylakoids which negatively interferes with photosynthesis.

## Conclusions

Considering the ultrastructural changes observed in this study it can be concluded that the size of chloroplasts and their ultrastructure are well suited as a stress marker for plants for abiotic and biotic stress. In this context the least costly and least labor-intensive process is to simply determine the number and size of chloroplasts on leaf sections of chloroplasts with the light microscope. For a more detailed evaluation, contents of starch and plastoglobules on-cross sections of chloroplasts are well suited. While starch completely disappeared, plastoglobules showed a massive increase of up to 19,617% on cross-sections of chloroplasts during dark-induces senescence. Thus, the absence of starch and the occurrence of large plastoglobules can also be regarded as a stress marker in plants which can be evaluated by TEM.
